# Iron economy in *Chlamydomonas reinhardtii*

**DOI:** 10.3389/fpls.2013.00337

**Published:** 2013-09-02

**Authors:** Anne G. Glaesener, Sabeeha S. Merchant, Crysten E. Blaby-Haas

**Affiliations:** ^1^Department of Chemistry and Biochemistry, University of California, Los AngelesLos Angeles, CA, USA; ^2^Institute of Genomics and Proteomics, David Geffen School of Medicine at the University of CaliforniaLos Angeles, CA, USA

**Keywords:** photosynthesis, transcriptome, ferredoxin, respiration, ferroxidases, photo-oxidative stress, acidocalcisome

## Abstract

While research on iron nutrition in plants has largely focused on iron-uptake pathways, photosynthetic microbes such as the unicellular green alga *Chlamydomonas reinhardtii* provide excellent experimental systems for understanding iron metabolism at the subcellular level. Several paradigms in iron homeostasis have been established in this alga, including photosystem remodeling in the chloroplast and preferential retention of some pathways and key iron-dependent proteins in response to suboptimal iron supply. This review presents our current understanding of iron homeostasis in Chlamydomonas, with specific attention on characterized responses to changes in iron supply, like iron-deficiency. An overview of frequently used methods for the investigation of iron-responsive gene expression, physiology and metabolism is also provided, including preparation of media, the effect of cell size, cell density and strain choice on quantitative measurements and methods for the determination of metal content and assessing the effect of iron supply on photosynthetic performance.

## Introduction

Although iron is relatively abundant in the earth's crust, inadequate access to this micronutrient often chronically limits photosynthesis in the ocean and on land. In oxygen-rich surface waters and neutral to alkaline soil, iron is present predominately in poorly soluble complexes, such as ferric oxides. The low bioavailability of iron complexes creates a major obstacle for photosynthetic organisms. Estimates indicate that iron limits phytoplankton growth in 40% of ocean waters (Moore et al., 2002) and that 30% of arable land is too alkaline for optimal iron uptake (Chen and Barak, 1982). This suggests that poor iron bioavailability causes consequential impacts on food chains, carbon sequestration and oxygen production.

Single-celled algae present a unique system for investigating how photosynthetic organisms respond to and cope with suboptimal iron nutrition. Specifically, in a laboratory setting, studying the subcellular response of plants to a deficient iron status can be complicated by the individual nutritional profile of each cell, tissue and organ type. Single-celled algae, on the other hand, such as the well-characterized green alga *Chlamydomonas reinhardtii* (referred herein as Chlamydomonas), are routinely grown in liquid cultures where the cell population and exposure to nutrients can be more homogenous. Chlamydomonas has been studied in the laboratory for well over 60 years as a convenient single-celled reference for understanding fundamental aspects of photosynthesis (Rochaix, 2002), including its specific relation to metal metabolism [reviewed in Merchant et al. (2006)]. The common laboratory strains are derived from a soil isolate, therefore, the natural environment of Chlamydomonas is close to that of land plants. Additionally, although the last common ancestor between Chlamydomonas and land plants such as Arabidopsis existed at least 700 million years ago (Becker, 2013), the photosynthetic apparatus are virtually identical. However, there are several metabolic differences; Chlamydomonas can oxidize acetate and exploits alternative bioenergetic routes such as hydrogen photoproduction and fermentation (Grossman et al., 2007).

This review focuses on our present understanding of iron nutrition in Chlamydomonas and on the preparation and use of iron-deficient and -limited media and common techniques to study physiology, gene expression and metabolism in Chlamydomonas. An effort is also made to point out caveats associated with these types of studies for all investigators to consider.

## Iron nutrition in chlamydomonas

Like land plants, algae have two especially iron-rich organelles, the chloroplast and the mitochondrion. Both organelles house numerous iron-dependent proteins whose functions are essential in the electron transfer pathways of the bioenergetic membranes in those compartments. In addition, iron is a component of many proteins involved in other essential processes such as reactive oxygen detoxification, fatty acid metabolism, and amino acid biosynthesis. Assuming a 1:1 stoichiometry of the electron transfer complexes [dimers for photosystem II (PSII) and the cytochrome *b*_6_*f* complex and a monomer for photosystem I (PSI)], linear electron flow from PSII to ferredoxin is estimated to require 30 iron ions (if plastocyanin is present, and 31 iron atoms if cytochrome *c*_6_ is present) (Blaby-Haas and Merchant, 2013). Again assuming 1:1 stoichiometry (monomers for complexes I, II, and IV, and a dimer for complex III), mitochondrial electron transport requires 50 iron atoms, over half contained within complex I (Xu et al., 2013). Although electron transfer in respiration appears to require more iron than does photosynthesis, the chloroplast is the dominant sink for iron in the oxygen-evolving plant cell, where this cofactor is concentrated in the abundant iron-dependent proteins of the thylakoid membrane.

The contribution of iron-dependent proteins in other cellular compartments to the cellular iron quota may be relatively small, but they are also essential for fitness or survival. These enzymes participate in DNA synthesis and repair [ribonucleotide reductase (cytosol) and DNA glycosylases (nucleus)], metabolite synthesis [cytochrome P450s (endoplasmic reticulum), aldehyde oxidase (cytosol), xanthine dehydrogenase (cytosol)], molybdopterin synthesis [Cnx2 (cytosol)], fatty acid metabolism [fatty acid desaturases (endoplasmic reticulum)] and reactive oxygen species detoxification [peroxidases (multiple compartments including peroxisome, Golgi, and cytosol)], just to name a few. Therefore, a delicate balance exists to ensure an appropriate amount of iron or iron-bound cofactor such as heme is present throughout the cell for the maturation of each iron-dependent protein.

**Figure 1:**
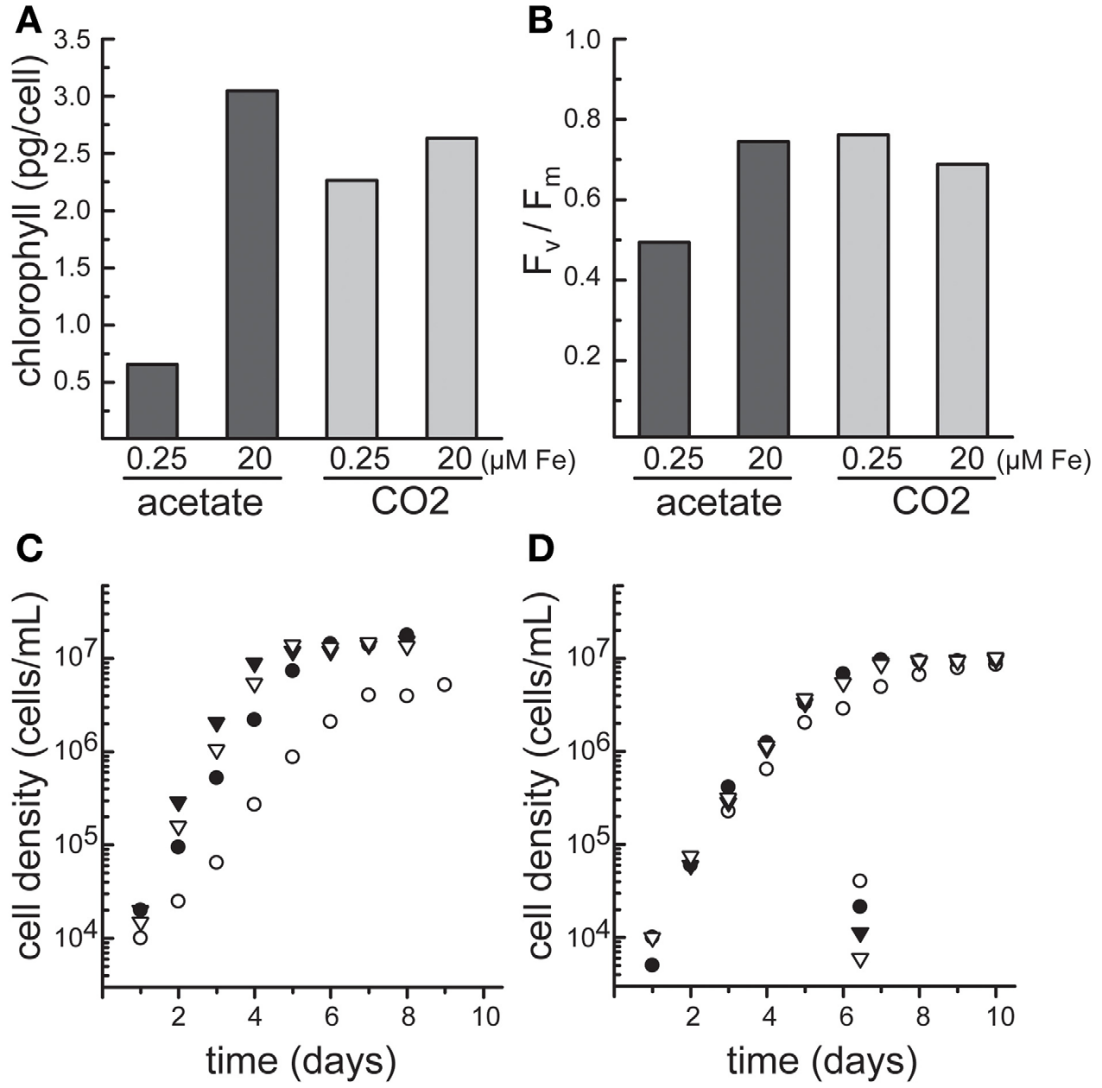
**The growth of Chlamydomonas CC-4532 in response to iron supplementation depends on the carbon source.** Cells were cultured in TAP for photoheterotrophic growth (acetate) or TP for photoautotrophic growth (CO_2_) using the revised trace metal supplement (Kropat et al., 2011) at 24°C and shaken at 180 rpm under continuous illumination of 70–80 μmol/m^2^s, and bubbled with sterile air. **(A)** Determination of chlorophyll content and **(B)** maximum quantum efficiency of photosystem II (*F*_*v*_/*F*_*m*_). Aliquots of cells grown photoheterotrophic (dark gray) and photoautotrophic (light gray) were removed from the culture during the logarithmic growth phase (~4 × 10^6^ cells/ml). **(C)** Photoheterotrophically and **(D)** photoautotrophically grown cells at 0.25 μM (open circles), 1 μM (filled circles), 20 μM (filled inverted triangles) and 200 μM Fe (filled inverted triangles) were counted with a hemocytomer for estimation of growth rates.

As a facultative photoheterotroph, Chlamydomonas can generate ATP from either photosynthesis or respiration depending on the presence of light and carbon source. This characteristic provides a unique and powerful experimental system to explore the effect of iron status on bioenergetic metabolism and vice versa. In particular, during the two major trophic states, photoautotrophic and photoheterotrophic, Chlamydomonas cells respond to iron status with acutely different physiologies (Figure 1). During the photoheterotrophic state, the cells are provided with light, CO_2_, and acetate. When iron becomes a limiting resource, competition for iron acquisition between the chloroplast and the mitochondria ensues. In response, the cell maintains respiration while decreasing the photosynthetic contribution to the bioenergetics of the cell, a phenomenon that may rely on preferential allocation of iron to the mitochondrion or recycling of iron from the chloroplast to the mitochondrion. In contrast, in the absence of acetate, photosynthetic activity is maintained, as seen by a less pronounced decrease in chlorophyll (Chl) content and maintenance of the maximum quantum efficiency of PSII (expressed by *F*_*v*_/*F*_*m*_) (Figures 1A,B) (Terauchi et al., 2010; Urzica et al., 2012).

To understand these phenomena and discover the underlying mechanisms, the study of iron homeostasis in Chlamydomonas is routinely performed in the context of four graded iron nutrition stages: excess, replete, deficient and limited. These states were delineated by the evaluation of phenotype and iron-responsive gene expression in response to controlled medium iron content (described in more detail in the following sections and summarized in Table 1).

**Table 1:** **Summary of the iron nutrition stages in Chlamydomonas distinguished by phenotype and sentinel gene expression**.

	**Photoheterotrophic**				**Photoautotrophic**		
	**Excess**	**Replete**	**Deficient**	**Limited**	**Replete**	**Deficient**	**Limited**
Fe in the media (μM)	200	20	1–3	0–0.5	20	1–3	0–0.5
Growth	Impaired only under light stress			Impaired			Slightly impaired
Fe atoms/cell (×10^7^)	50–100	8–20	2–12	1–4	14	6–9	3
*FOX1* expression	Basal	Basal	Up	Up	Basal	Up	Up
Chl content (pg/cell)		2.3–2.5	1.8–2	0.6–1.2	2.6	2.6	1.8

**Figure 2:**
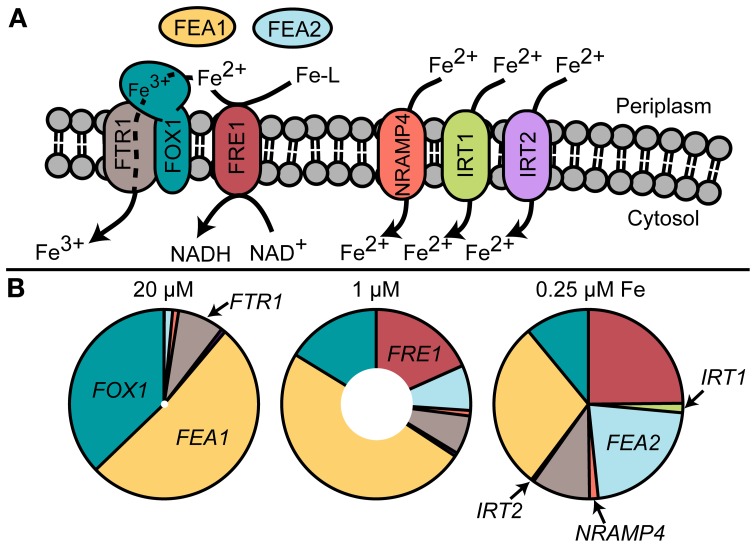
**(A)** Known and putative iron uptake pathways in Chlamydomonas and **(B)** transcript abundance of uptake components estimated by RNA-Seq (Urzica et al., 2012). The inner white circles represent total transcript abundance relative to 0.25 μM iron.

**Figure 3:**
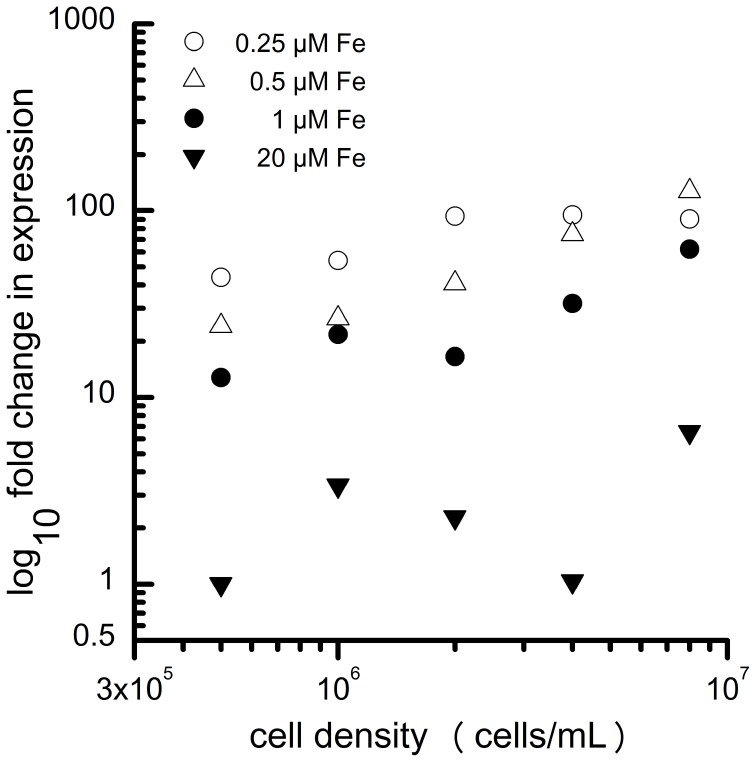
**Expression of *FOX1* depends both on extracellular iron content (0.25 μM, open circles; 0.5 μM, filled triangles; 1 μM, filled circles; 20 μM, filled inverted triangles) and the number of cells per mL.** The increased expression of *FOX1*during growth of the batch culture likely reflects the reduced Fe availability on a per cell basis. Adapted from Urzica et al. (2012).

Specifically, components of the iron-uptake pathway are routinely used as sentinel genes for iron status. In contrast to land plants, the main iron uptake pathway in Chlamydomonas (based on transcript and protein abundance) is the fungal-like ferroxidase-dependent ferric transporter complex consisting of FOX1 (the ferroxidase) and FTR1 (the permease) (Figure 2). The copper-containing enzyme FOX1 catalyzes the oxidation of Fe(II) to Fe(III), similar to the yeast and human enzymes, Fet3p and ceruloplasmin, respectively (Herbik et al., 2002; La Fontaine et al., 2002). FOX1 is presumed to form a complex with the permease FTR1, which transports the ferric iron provided by FOX1 into the cytosol (Terzulli and Kosman, 2010). *FOX1* expression responds quickly to changes in iron nutrition ahead of any observable effects on physiology and thus provides a convenient and robust marker for iron status (Figure 3).

It should be noted that the following iron nutrition stages are described for cells grown photoheterotrophically (light and acetate). The same stages can be described for phototrophic cells (light and CO_2_), but the iron concentration thresholds are distinct, as is the cellular response to those concentrations. These stages are also likely distinct for cells grown purely heterotrophically (dark and acetate), but this phenomenon has not yet been systematically studied.

### Stage 1: iron excess

In the iron excess stage (200 μM medium iron content), the cells will over-accumulate iron, as compared to the iron-replete stage (Long and Merchant, 2008; Terauchi et al., 2010). This observation is in contrast to the copper-excess situation where the cells only take up as much copper as needed despite over 500-fold excess in the medium (Page et al., 2009). Although the culture does not display any visible phenotype associated with iron excess, the cells are unable to grow at high photon flux density (500 μmol/m^2^s at the surface of a petri dish) (Long and Merchant, 2008). This result suggests that the higher iron content of the cells, which is 2- to 5-fold higher than in replete cells, exacerbates damage caused by photo-oxidative stress and that there may be a pool of reactive iron in the chloroplast. In contrast to mammals and higher plants, iron excess does not affect the abundance of ferritin in Chlamydomonas, leading to the conclusion that ferritin is not the major iron storage molecule in the cell (Busch et al., 2008; Long et al., 2008). For Chlamydomonas, there are no known mechanisms for iron export, presumably because this high iron concentration is not typically experienced in nature and there is no need to establish pathways to deal with the excess.

Based on Mössbauer spectroscopy of iron-replete cells, Semin et al. found that Chlamydomonas cells contain an unusally high amount of ferrous iron. The authors have postulated that instead of typical iron storage proteins such as ferritin or siderophores, which would bind ferric iron, iron is stored in vacuoles as in yeast (Semin et al., 2003). This compartment may in fact be the acidocalcisome, which is an acidic, calcium- and polyphosphate-rich, membrane-bound, lysosome-related organelle (Ruiz et al., 2001; Docampo et al., 2005). Indeed, in the green alga *Dunaliella salina* and the red alga *Cyanidium caldarium*, intracellular iron is found in acidic vacuoles (Paz et al., 2007; Nagasaka and Yoshimura, 2008).

### Stage 2: iron replete

The standard growth medium for Chlamydomonas contains 18–20 μM iron (depending on the trace supplement) (Hutner et al., 1950; Kropat et al., 2011). In both trace mixes, iron is supplied as an EDTA chelate, which is used to maintain iron in solution, thereby facilitating uptake. However, it should be mentioned that for the Hutner's trace mix, 18 μM iron is the concentration of iron added to the mix, but because of ferric iron precipitation during preparation, there is batch-to-batch variation, and the concentration of soluble iron may be closer to 10 μM (Kropat et al., 2011). Recently, a revised trace metal supplement for Chlamydomonas was formulated (Kropat et al., 2011). Using the cellular content of metals during growth in the replete condition, the trace metal composition was adjusted to supply a 3-fold excess of each nutrient. Some components not utilized by Chlamydomonas found in the Hunter's trace mix were removed, such as cobalt and boron, and the concentrations of other metals, such as zinc and manganese were reduced. Several benefits are apparent with use of the new formulation. The cells have an increased growth rate, and the supplement is more stable and less time consuming to prepare. Instead of combining the mineral nutrients into a single stock, each component is stored separately and added freshly while preparing media. In terms of iron nutrition, the greatest benefit of the revised mix is the absence of iron precipitation (over a 5 years period).

As the culture reaches stationary growth, the cells in replete medium have consumed (depending on the strain) 10–30% of the medium iron content (Kropat et al., 2011; Page et al., 2012). This “luxury” consumption is characterized by basal low level expression of the genes encoding the high-affinity FOX1/FTR1 ferric iron transporter and negligible expression of the putative secondary iron transporters NRAMP4, IRT1, and IRT2 (Urzica et al., 2012). In the replete stage, Chl concentration has been measured at roughly 2.5 pg/cell Chl (Moseley et al., 2002; Page et al., 2012; Urzica et al., 2012). However, the ratio of Chl per cell or Chl per protein is affected by the incident illumination and the strain genotype.

### Stage 3: iron deficient

As the iron content of the medium is reduced to around 1–3 μ M, the cells begin to experience iron deficiency. The expression of iron uptake pathways is dramatically induced. Increased abundance of FOX1 at either mRNA or protein levels is commonly used as a sentinel marker for iron-deficiency. Well characterized primers for qPCR and antibodies for immunoblot analysis are available (Herbik et al., 2002; La Fontaine et al., 2002; Allen et al., 2007a). At this concentration of iron supply, the cells are usually not chlorotic and photosynthetic complex abundance is generally not affected (Moseley et al., 2002). Of course, depending on genotype, in some strains the lower end of this concentration range may result already in symptoms of limitation (see below), such as marginal chlorosis and a very mild impact on abundance of photosynthetic complexes. In general, this stage is differentiated from the iron-limited state by the lack of a growth phenotype.

Despite the absence of chlorosis in the iron-deficient state, spectroscopic measurements revealed some changes within the chloroplast. Specifically, fluorescence rise and decay kinetics (Kautsky curves) indicate that re-oxidation of the plastoquinone (PQ) pool is slower in the iron-deficiency situation (Moseley et al., 2002). This has been attributed to some loss of function of iron-containing electron transfer complexes downstream of the PQ pool, such as the cytochrome *b*_6_*f* complex, PSI or ferredoxin. The relatively exposed [4Fe-4S] clusters of PSI are prime candidates.

Structural changes in the PSI-LHCI complex accompany iron deficiency, presumably to compensate for reduced PSI function. These modifications to the complex result in reduced energy transfer from the accessory antenna to the reaction center (Moseley et al., 2002). This results both from dissociation of PsaK, a connector between PSI and LHCI, from PSI and proteolysis of individual Lhca subunits in LHCI (Ben-Shem et al., 2003; Naumann et al., 2005). The signal transduction pathway leading to the structural modification of PSI-LHCI is not known, nor is the mechanism of PsaK and Lhca degradation. Mixing experiments indicate that degradation does result from induced proteolysis (Moseley et al., 2002). One idea that has been put forward regarding sensing of iron status by PSI is that it may occur by occupancy of the Chl binding sites in PsaK. If these sites are low affinity, they may be sensitive indicators of flux through the Chl biosynthetic pathway, which is dependent on iron at the rate-limiting step catalyzed by aerobic cyclase (Tottey et al., 2003).

Based on genome-wide expression profiling using RNA-Seq methodology, 78 genes displayed at least a 2-fold difference in transcript abundance between this stage (1 μ M Fe) and the replete stage (Urzica et al., 2012). Since many organisms occupy a niche that allows them to survive in just barely sufficient iron, the transcriptome of this state is clearly relevant to the impact of marginal iron nutrition on crop yields and primary productivity. The two largest functional groups (for this particular dataset) represented in the iron-deficient stage encode proteins with known or predicted function in metal transport (17%) and the redox/stress response (25%). This includes the upregulation of genes involved in iron transport, like the ferric reductase *FRE1*, the high-affinity iron-uptake system (comprising *FOX1* and *FTR1*), the transcripts for the algal-specific proteins FEA1 and FEA2, and the putative secondary iron transporters *NRAMP4* and *IRT2*. The expression of a Mn-dependent superoxide dismutase is also highly induced. The transcripts of only four genes were significantly reduced in abundance during this condition. One of these is *FDX5*, an anaerobically-induced chloroplast-targeted ferredoxin which contains a [2Fe-2S] cluster (Jacobs et al., 2009). The reduction of *FDX5* transcript abundance may serve to spare iron.

The large proportion of redox/stress-related transcripts at this stage of iron nutrition, which is visually asymptomatic, may reflect an anticipatory response to incipient stress associated with changes in light harvesting and reduced rate of electron transfer downstream of the PQ pool. Additionally, these transcripts may be related to compromised PSI, which can produce superoxide by the photoreduction of oxygen. Solvent-exposed iron-sulfur clusters are particularly sensitive to superoxide and are consequently destroyed releasing ferric iron. If not immediately chelated, the free iron can react with hydrogen peroxide creating the hydroxyl radical, a highly cytotoxic molecule, which cannot be detoxified enzymatically. Indeed, expression of the two ferritin genes in Chlamydomonas is induced during iron deficiency. The ferritins localize to the chloroplast, but only ferritin1 appears to increase in abundance in response to iron-deficiency (Busch et al., 2008; Long et al., 2008). Although ferritins are typically regarded as iron storage complexes, neither of the two Chlamydomonas complexes appears to contain significant amounts of iron when cells were grown in iron-deficient medium, leading to the conclusion that ferritin buffers instead of stores iron liberated within the chloroplast. As a unicellular organism, iron homeostasis is focused on distribution at a sub-cellular level, including partitioning to the mitochondria vs. the chloroplast, and this may account for the unique response of ferritin within the chloroplast.

### Stage 4: iron limitation

As the iron content of the medium is reduced below about 0.5 μM, cells enter the iron-limited stage, where cell growth is inhibited due to limiting nutritional supply of iron. Although the iron transport pathways are still highly expressed, and at a higher level than in the deficiency state (Figure 3), the cells are markedly chlorotic, corresponding to a decrease in Chl of about 2- to 4-fold, and the growth rate is reduced (Figure 1). Concurrently, multiple iron-containing proteins in the chloroplast are reduced in abundance. These include PSI (12 iron atoms), the cytochrome *b*_6_*f* complex (12 iron atoms) and ferredoxin (2 iron atoms), and as a result, iron-limited cells exhibit a severe block in photosynthesis (Moseley et al., 2002; Page et al., 2012; Urzica et al., 2012). The noticeable loss of iron-bound photosynthetic complexes is generally only seen for cells grown in media containing acetate as carbon source, whereas photoautotrophically grown cultures maintain photosynthetic performance and retain these complexes for an extended period of time during iron-limitation. During photoheterotrophic iron-limited growth, subunits of respiratory complexes localized in the mitochondria change in abundance, as shown by immunoblot analysis and comparative quantitative proteomics (Naumann et al., 2007; Terauchi et al., 2010). The abundance of complex I subunits is decreased, whereas subunits of complexes III and IV increase. In the absence of acetate, abundances of respiratory complexes remain unchanged during iron-limitation.

Urzica et al. found a large number of genes with increased transcript abundance (at least 2-fold difference; 2050 genes) during this stage (0.25 μ M) relative to iron replete, underscoring the stress induced by iron limitation (Urzica et al., 2012). As seen in the iron-deficient dataset, the most dramatically increased transcripts are those involved in iron transport and those encoding Mn-dependent superoxide dismutase (Urzica et al., 2012). The large number of differentially abundant transcripts during this condition also highlights the value of studying the transcriptome of the iron-deficient state, where only 78 RNAs are significantly changed in abundance. It is more likely that these 78 RNAs include the direct targets of iron nutrition acclimation rather than secondary stress responses. Indeed, several transcriptome studies of iron-starved land plants have had to contend with large sets of transcripts with changed abundance (Thimm et al., 2001; Zheng et al., 2009). The mechanical stress of transferring plants from iron-sufficient to -deprived medium (Buckhout et al., 2009) and the cumulative response from different cell types and tissue often obscures the primary iron responses.

## Common methods for studying iron nutrition in chlamydomonas

Three basic techniques are generally used to generate poor iron nutrition in the laboratory. The first is to limit the available iron in the medium by chelators. For work with yeasts like *S*. *cerevisiae* and *Schizosaccharomyces pombe*, iron-chelators such as 2,2′-dipyridyl and bathophenanthroline disulfonic acid, are often used to generate a state of poor iron nutrition *in vivo* (Eide et al., 1996; Pelletier et al., 2005; Mercier et al., 2006; Jo et al., 2009). Some studies with plants like *Arabidopsis thaliana* have combined the use of chelators like ferrozine with the strategy of creating iron deficiency by omitting iron from the media (Vert et al., 2002; Lanquar et al., 2005; Yang et al., 2010). For Chlamydomonas, some studies have used the chelators ferrozine and ethylenediamine-N,N′-bis(2-hydroxyphenyl)acetic acid (EDDHA) (Xue et al., 1998; Weger, 1999; Weger and Espie, 2000; Rubinelli et al., 2002). However, when using chelators or evaluating studies that solely used chelators to achieve iron depletion, several caveats should be kept in mind. As with all metal chelators, these molecules are not specific to iron and will bind other metal ions (Kroll et al., 1957; Stookey, 1970), possibly leading to observations not specifically caused by iron depletion. In addition, the cells may be able to compete with the chelator leading to slower iron uptake, which may affect how the cells respond as compared to an actual omission of iron from the media. In some ways, however, the use of chelators could be a more appropriate approach to the study of iron homeostasis, because in most environments poor iron nutrition is due to competition between iron uptake pathways and natural ligands in contrast to the absence of iron.

The second technique is to limit intracellular iron by using an iron-transport mutant. This approach has not been routinely employed for work with Chlamydomonas but is commonly used in other organisms such as yeast and land plants. In *A. thaliana*, a mutant of the iron transporter *IRT1* was used to elucidate the role of iron-nutrition on lateral root development (Giehl et al., 2012), gene expression (Wang et al., 2007), and circadian rhythm (Hong et al., 2013; Salomé et al., 2013). In the yeast *S. cerevisiae*, the *fet3 fet4* double mutant, lacking both the high and low affinity iron transport pathway, is very sensitive to poor iron nutrition (Dix et al., 1994). This mutant has been used to study siderophore uptake (Lesuisse et al., 1998) and to investigate the relationship between iron homeostasis and an anti-malaria drug (Emerson et al., 2002). Although the use of iron transport mutants is a convenient method to achieve cellular iron depletion, iron will likely enter the cell via other routes and uptake of other metal ions can be affected in the mutant.

The third and preferable approach is to control the amount of iron added to the medium, described in more detail in section Preparing Media. Of course, in each of these cases, one has to worry about whether deficiency in one metal affects the intracellular concentration of other metal ions. For instance, non-selective transporters may be induced, which will inadvertently bring in multiple metal ions. The cell may purposefully change the concentration of other metals as seen for iron and manganese and for zinc and copper levels in Chlamydomonas (Allen et al., 2007b; Malasarn et al., 2013). Therefore, it is imperative to measure all intracellular metal concentrations during iron nutrition experiments (see section Metal Measurement).

### Preparing media

Chlamydomonas is routinely cultured in a simple, defined medium where the concentration of metal ions can be selectively controlled. Popular media include Sueoka's high salt medium (HS or HSM) and Tris-phosphate medium (TP), both of which can be supplemented with the carbon source acetate (HSMA and TAP, respectively). The largest difference between the two media is the presence of Tris(hydroxymethyl)aminomethane (Tris) in TP and a roughly 14-fold higher concentration of potassium and phosphate in HSM. TAP/TP has a higher capacity to buffer pH changes in the culture compared to HSMA/HSM, which should be considered when choosing a medium, since the pH of the medium affects the availability of iron. Both types of media are routinely used for iron nutrition studies in Chlamydomonas. Indeed, TAP and HSM are generally used to compare photoheterotrophic and photoautotrophic growth. However, because the compositions of the two media are different, using TAP and TP or HSMA and HSM is preferred.

**Figure 4:**
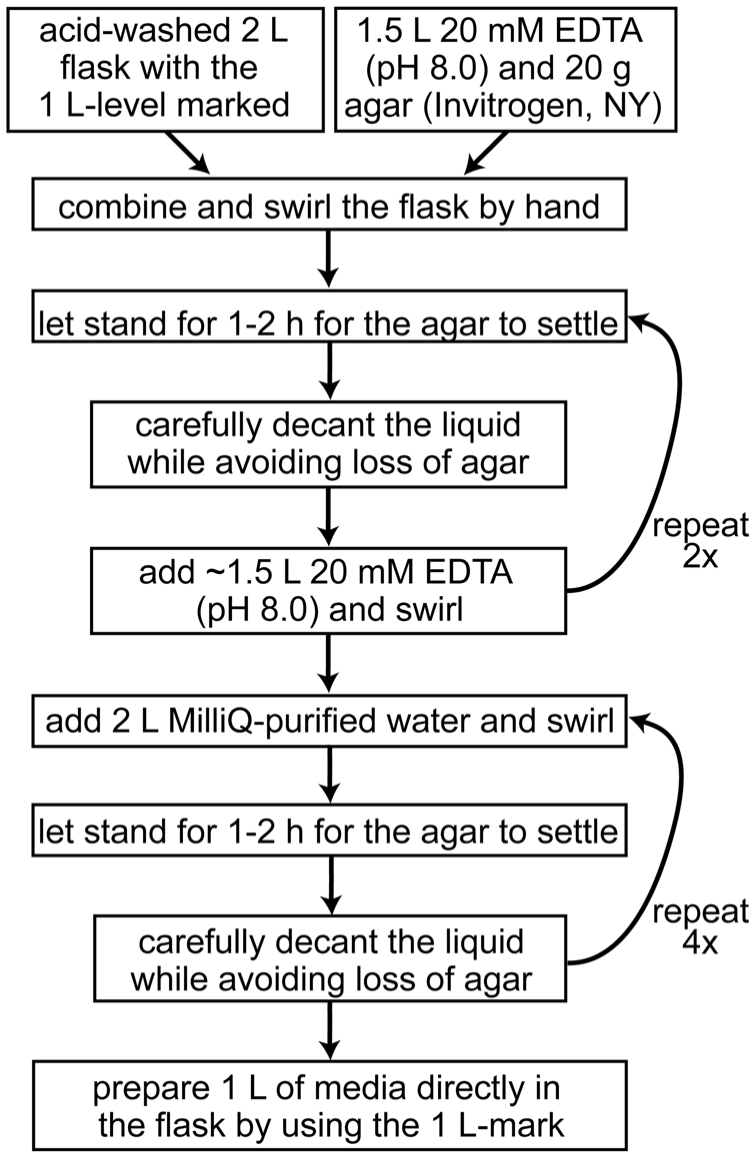
**Workflow for the preparation of iron-depleted solid growth media for Chlamydomonas**.

Because of high metabolic demand for iron, iron depletion in laboratory media is relatively easy to accomplish in Chlamydomonas. However, several precautions should be followed to ensure that the amount of iron in the medium is tightly controlled and the experiments are therefore reproducible, i.e., the only iron present is that which is consciously added. Most recent iron metabolism studies of Chlamydomonas employ the media preparation methods detailed in (Quinn and Merchant, 1998) for achieving copper deficiency. In summary, the use of clean glassware and plasticware is of paramount importance. All culture flasks and reusable plasticware are rinsed at least twice with 6 N HCl to displace metal ions and rinsed six times with MilliQ-purified water to remove the HCl. High-purity chemicals are used to make iron-free stock solutions, which are stored in metal-free plasticware. Certificates of analysis specifying the trace metal composition are generally available before purchase of the stock chemicals and can be used to estimate the amount of contaminating metals in the prepared medium. These chemicals should be kept separate from other laboratory chemicals to avoid accidental metal contamination. The preparation of solid iron-deficient media requires washing of the agar with EDTA to remove contaminating metal ions (Figure 4). At all times, effort should be made to avoid contamination with metals (wear gloves, no metal spatulas and protection from dust). Ideally, iron-deficient media prepared in glass flasks should be used immediately. It is recommended that the media not be stored for more than a couple of days. Even though the flasks are acid washed, there will be residual metal ions remaining in the glass, which will leach into the medium over time (Cox, 1994).

#### The use of pH

Alkaline pH is commonly employed in iron nutrition experiments with soil-grown plants that rely on acidification and reduction to solubilize iron (i.e., Strategy I plants). This strategy has not been generally applied to Chlamydomonas, because of the ease with which the iron concentration in the medium can be controlled.

### Strains

**Figure 5:**
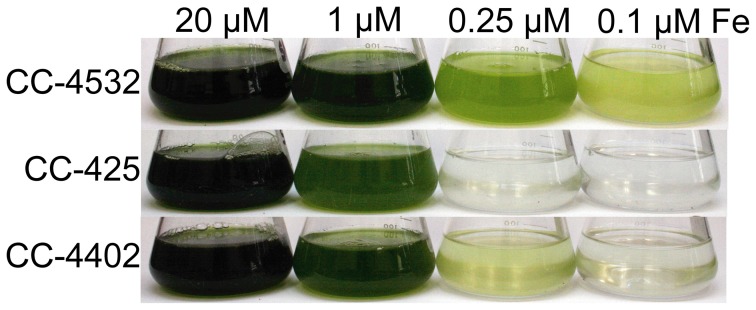
**Growth of different Chlamydomonas strains in response to iron supply.** CC-4532 and CC-4402 are cell wall-containing strains, whereas CC-425 represents a cell wall-less strain. Cultures were inoculated at 1 × 10^4^ cells/mL and grown for 10 days in TAP (Tris-acetate-phosphate) supplemented with iron to the indicated concentrations at 24°C and shaken at 180 rpm under constant illumination of 60–80 μmol/m^2^s.

The genotype of each “wild-type” Chlamydomonas strain can have a noticeable effect on a strain's tolerance to poor iron nutrition. Most of the commonly used laboratory strains appear to be descendants of a cross between two divergent spores from the original isolate, and the resulting progeny may have one or the other of two distinct haplotypes at any given locus (Gallaher et al., manuscript in preparation). In practice, this means that even closely related strains, such as CC-4402 and CC-4532, can have thousands of SNPs relative to each other, and will exhibit observable phenotypic differences (Figure 5).

The largest difference between strains in terms of iron nutrition is the presence or absence of a cell wall. Some researchers have chosen to employ a cell wall-less strain in their studies to avoid overestimation of iron uptake rates caused by iron bound to the cell wall (Lynnes et al., 1998). However, it was later found that two key components of the iron assimilation pathway are soluble proteins secreted to the periplasmic space between the plasma membrane and cell wall. Expression of the genes encoding these algal-specific proteins, *FEA1* and *FEA2*, is significantly induced during iron deficiency, and in a cell wall-less strain, these proteins are lost to the medium (Allen et al., 2007a). Although we do not know the function of these periplasmic proteins, it is hypothesized that they may bind iron and may serve to concentrate iron (whether Fe(II) or Fe(III) is not known) in proximity to the plasma membrane assimilatory transporters. A significant consequence of the loss of the FEA proteins into the medium is increased sensitivity of these strains to iron depletion (strain CC-425 in Figure 5).

### Cell size

Metal and Chl content are commonly reported on a per cell basis for Chlamydomonas. This convention can complicate comparisons of iron homeostasis studies employing different Chlamydomonas strains or cells grown under different growth regimes as cell size can vary between laboratory strains and growth conditions. Cell size was reported to be affected by trophic status (Terauchi et al., 2010), nutrient stress (Zhang et al., 2002; Kropat et al., 2011), CO_2_ concentration (Vance and Spalding, 2005), HSM compared to TAP media (Fischer et al., 2006), light quality (Murakami et al., 1997), light intensity (Matsumura et al., 2003) as well as during the cell cycle (Umen, 2005). Additionally, cell size can vary between mutant and parent. For instance, a ferritin knockdown mutant was found to be almost 2-fold bigger in size than the wild-type strain (Busch et al., 2008).

### Cell density

In general, it is advisable to control for cell density rigorously during sampling of cells for molecular analyses so that external iron concentration can be used as a proxy for intracellular iron content. A typical laboratory culture of Chlamydomonas will consume iron equivalent to about 3 μM as it goes from inoculation to stationary phase (Page et al., 2012). Therefore, in medium containing iron in this concentration range (or lower), expression of the nutritional iron regulon is dependent on the cell density (Figure 3). As the cells in culture divide, the ratio of medium iron per cell decreases, and expression of genes involved in iron assimilation steadily increases (Urzica et al., 2012). This relationship is not evident in medium containing excess iron (see 20 μM samples in Figure 3) where the *FOX1* marker gene is expressed at a very low basal level. Interestingly, in fully replete media, during logarithmic phase, the demand for intracellular iron exceeds the capacity of the iron uptake and metabolism pathway, resulting in transient iron deficiency (Page et al., 2012).

### Metal measurement

Element quantification based on inductively coupled plasma in combination with mass spectrometry (ICP-MS) or optical emission spectroscopy (ICP-OES) enables investigators to measure multiple metals within a sample. For mass spectrometry based detection, the plasma is used to ionize the atoms, which are then separated on the basis of their mass to charge ratio (Husted et al., 2011). For detection by OES, atoms in the sample are excited by argon plasma and emit light at their characteristic wavelengths, which is used to identify the elements in the sample (Hou and Jones, 2000).

Theoretical detection limits of ICP-MS for most elements are at about 10 parts per trillion (ppt). When measuring complex biological samples, one has to consider matrix effects caused by the cell material, and measuring a standard curve in the cell paste is recommended to verify that matrix effects are negligible. The theoretical limit for ICP-OES is typically two- to three orders of magnitude higher than for ICP-MS with most elements detected at 1–10 parts per billion (ppb). Both detection methods have a linear dynamic detection range over several orders of magnitude (usually a linear dynamic range of 10^6^ to 10^7^) (Pröfrock and Prange, 2012).

Yet the use of ICP-MS for iron quantification has a limitation with respect to sensitivity when argon is used to generate the plasma. Polyatomic interferences, mainly due to argon oxide, have the same mass as the most abundant Fe isotope, ^56^Fe, and prohibit accurate measurement (Vogl et al., 2003). Therefore, in order to measure the iron content in a sample, a less abundant isotope of iron (^57^Fe) can be measured. Another possibility is to use helium or hydrogen as a collision gas, which reduces the occurrence of interfering polyatomic complexes (Niemelä et al., 2003). However, depending on the tuning of the collision gases, the background equivalent concentration (BEC) can be as high as 2 ppb. Therefore, to be able to measure accurate iron concentrations, the concentration of the sample has to be above the background level. Newer ICP-MS instruments utilize a combination of collision/reaction cell technologies with quadrupole or octupole mass analyzer to minimize interferences even further (Yip and Sham, 2007; Cvetkovic et al., 2010).

**Figure 6:**
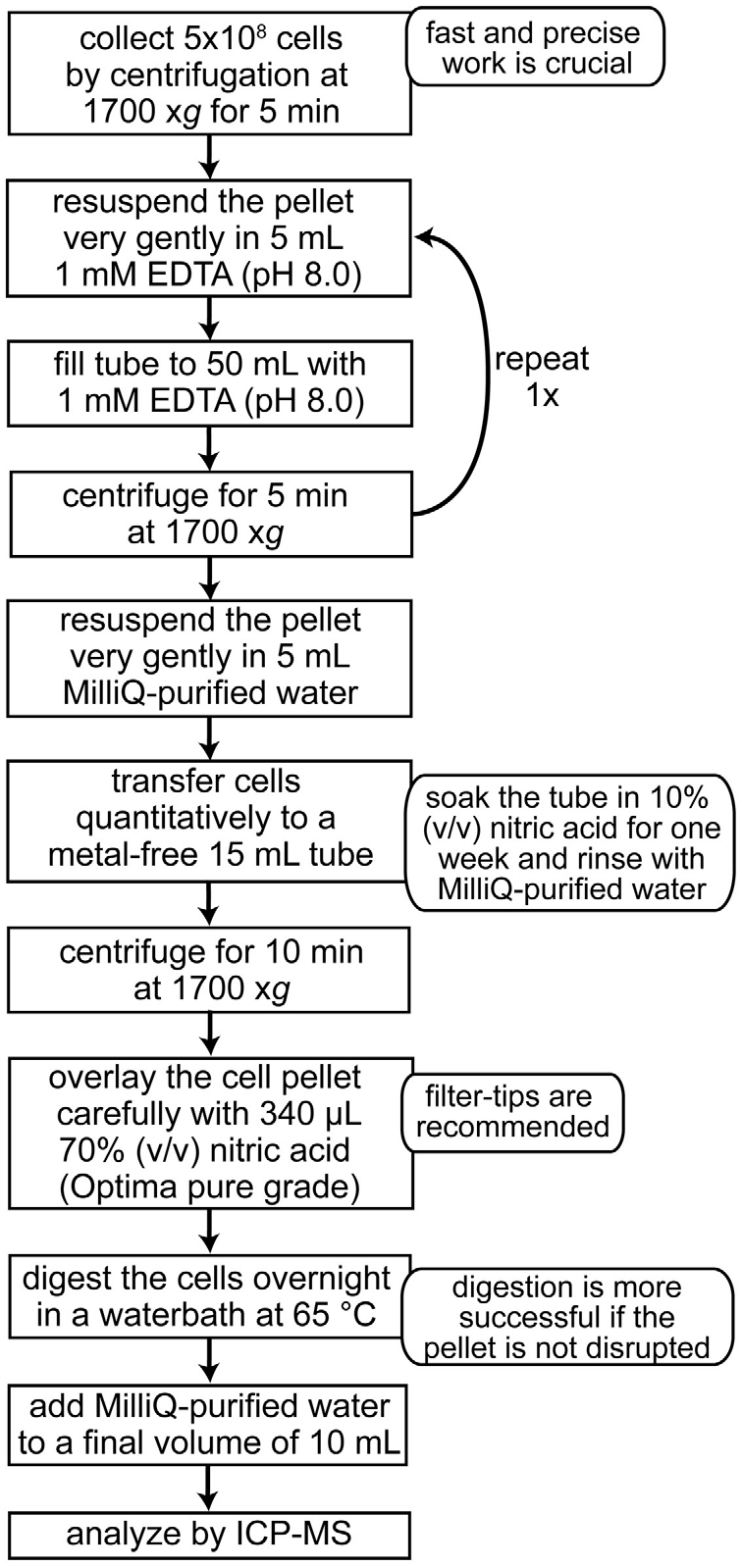
**Sample preparation of Chlamydomonas cells for metal analysis by ICP-MS**.

Because of the wide dynamic range, both the concentration of iron in the medium (1116 ppb) and in the cells can be quantified by either ICP-MS or ICP-OES. ICP-MS measurements (Agilent 7500) for different Chlamydomonas strains grown photoheterotrophically in iron-replete (20 μM) media range between 5 and 40 × 10^7^ Fe atoms per cell (corresponding to 46 and 371 ppb, when digested cell paste equivalent to 1 × 10^7^ cells/mL is measured) (Kropat et al., 2011). The cellular iron content of the strain CC-125 is reduced from 12–25 × 10^7^ Fe atoms per cell to about 5 × 10^7^ Fe atoms per cell within 24 h of transfer from iron replete to iron-minus medium (Page et al., 2012). This iron content corresponds to about 110–230 ppb for iron-replete and 46 ppb Fe for iron starved cells (for sample preparation, see Figure 6).

As mentioned in section Cell Size, the cell size can vary between strains and growth conditions, and the calculation of metal content on a per cell basis, even though broadly used, is not ideal for comparisons between experiments conducted with different strains or under different growth conditions. Normalization based on the amount of other elements measured from the same sample would be desirable, but this normalization method should also be performed with caution, and efforts made to ensure that the chosen element's concentration does not change between test conditions. For instance, phosphorous content is commonly used to normalize the metal content between samples. However, Chlamydomonas manganese-deficient cells contain less phosphorous than replete cells (Allen et al., 2007b). The measurement of total organic carbon for normalization purposes may be a likely alternative.

### Methods for monitoring photosynthetic parameters

#### Chlorophyll assay

Interveinal chlorosis is a classic symptom of poor iron nutrition in plants and is indeed a convenient indicator of iron status in agriculture (Mengel, 1994). It was originally attributed to the impact of low iron supply on the function of an iron-dependent step in Chl biosynthesis (Brown, 1956), but there may also be programmed degradation of Chl-containing proteins (Spiller et al., 1982; Moseley et al., 2002).

Unlike in land plants, the extent of chlorosis is dependent on the growth mode of Chlamydomonas cultures—photoheterotrophic vs. photoautotrophic. As a result, chlorosis by itself is not an absolute indicator of iron status. As mentioned above, the photosynthetic machinery is maintained longer in iron-limited cells grown in the light without acetate, while in the presence of acetate, degradation is apparent at early stages of suboptimal iron nutrition (Figures 1A,B) (Terauchi et al., 2010; Urzica et al., 2012).

**Figure 7:**
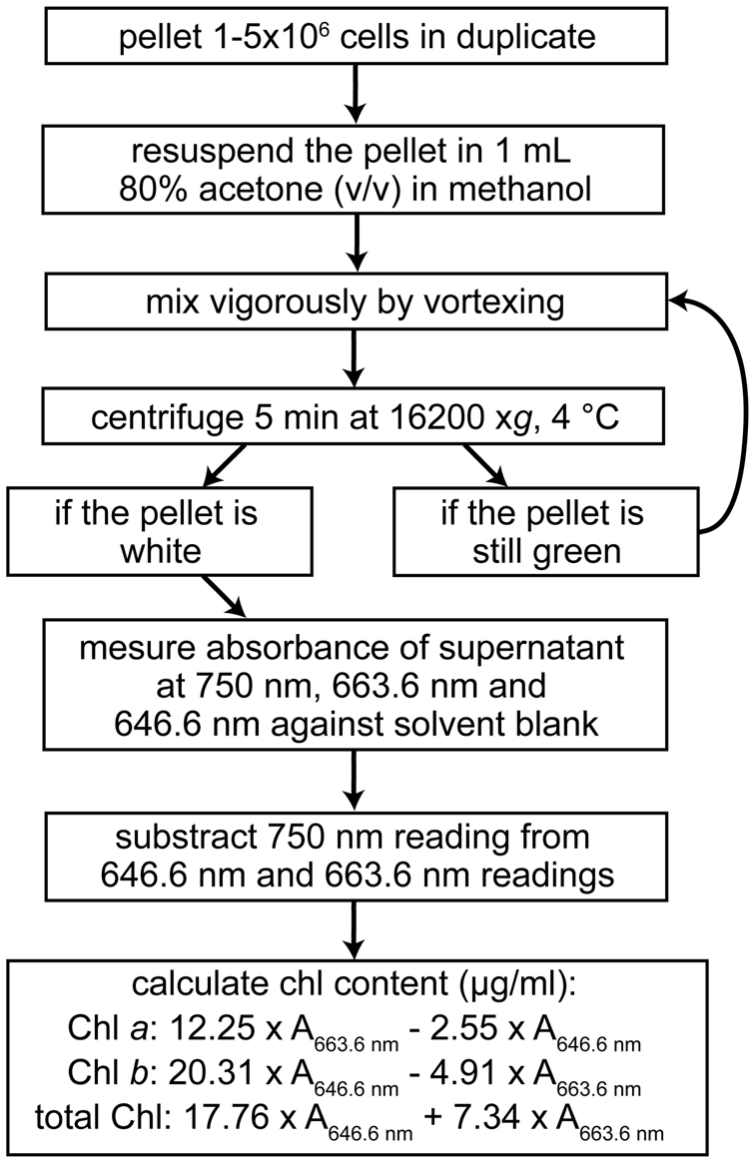
**Workflow for the spectrophotometric determination of the chlorophyll content of Chlamydomonas cells.** Calculations are based on Porra et al. (1989).

For the measurement of Chl content, quantitative extraction of all Chl molecules from the cell is important. For Chlamydomonas this is routinely achieved with 80% (v/v) acetone in methanol (Figure 7) (Porra et al., 1989; Moseley et al., 2000). Chl *a* and *b* concentrations are estimated according to the method of Porra et al. (1989). Even though the extinction coefficients in that method were determined for 80% acetone in aqueous solution, the estimation is accurate enough for comparing strains or conditions. In addition, extraction of Chl with 100% methanol has also been used in Chlamydomonas (Lynnes et al., 1998).

#### Chlorophyll fluorescence

Chlorophyll fluorescence is a fast, non-destructive way of characterizing the photosynthetic status of a cell. Absorbed photons generate excited Chls that can participate in photochemistry, dissipate the absorbed energy as heat (non-photochemical quenching) or return to the ground state accompanied by emission of light, referred to as Chl fluorescence. Since these individual fates of the excited state are mutually exclusive, the measurement of Chl fluorescence can be used to assess the efficiency of photochemical and non-photochemical quenching in the reaction center [the reader is encouraged to see (Maxwell and Johnson, 2000) and (Baker, 2008) for more details].

To probe the effect of iron nutrition on photosynthetic performance, Moseley et al. measured Chl fluorescence induction and decay kinetics of Chlamydomonas cells grown photoheterotrophically under different iron nutrition stages. The cells were dark-adapted for at least 5 min and exposed to actinic light with a photon flux density of ~60 μmol/m^2^s, using an open FluorCam detector to record the resulting fluorescence emission (Moseley et al., 2002). During dark-adaptation, the absence of photons and the consequent absence of electron output from PSII causes the Q_A_ pool (the primary quinone electron acceptor of PSII) to become maximally oxidized. When these cells are exposed to actinic light, the Kautsky effect is observed: a fast increase in Chl fluorescence due to initial reduction of Q_A_ followed by a slow decay due to its subsequent re-oxidization. Photoheterotrophic iron-deficient Chlamydomonas cells (1 μM Fe in the growth medium) display a slower fluorescence decay rate compared to iron replete cells, indicative of a reduced rate of Q_A_ re-oxidation, possibly caused by impaired function of iron-containing complexes downstream of the PQ pool (Moseley et al., 2002). The slower decay rate is exacerbated in iron-limited cells (0.25 μ M Fe in the media) and accompanied by a slower rate of Q_A_ reduction and a higher fluorescence yield suggesting impairment of PSII in addition to downstream electron transfer (Moseley et al., 2002).

The measurement of Chl fluorescence can also be used to determine photosynthetic parameters which help to determine the photosynthetic status of a cell. The ratio *F*_*v*_/*F*_*m*_ (maximum quantum efficiency of PSII) is a frequently used parameter, its value is relatively constant but decreases in stressed cells. Using a Hansatech FMS2 pulse-modulated fluorometer, Terauchi et al. measured the fluorescence of Chlamydomonas cells grown in the presence of different iron concentrations under both photoautotrophic and photoheterotrophic conditions. Cells were dark-adapted for 15 min and subsequently filtered onto 13-mm diameter Milipore AP20 glass fiber filters prior to measurement (Terauchi et al., 2010). *F*_*v*_ is defined as the difference between *F*_*m*_ and *F*_0_, with *F*_0_ as the minimum fluorescence of the sample after dark-adaptation and *F*_*m*_ the maximal Chl fluorescence after illumination with actinic light (Baker, 2008). Insufficient iron nutrition during photoheterotrophic growth of Chlamydomonas cells results in decreased *F*_*v*_/*F*_*m*_ from 0.7–0.74 in iron-replete to 0.67 and 0.54 in 0.2 and 0.1 μM Fe in the growth media, respectively. This decrease was not observed during iron-limitation in photoautotrophic growth conditions, indicating maintenance of photochemistry during this trophic growth regime (Figure 1B) (Terauchi et al., 2010).

#### 77 K fluorescence

Low-temperature (77 K) fluorescence emission spectra of whole Chlamydomonas cells or isolated thylakoid membranes are generally composed of major fluorescence bands around 685 and 715 nm, which correspond to fluorescence emitted from Chl in light-harvesting antennae connected with PSII (685 nm) and connected with PSI (715 nm) (Murata, 1968). This technique can be used to analyze changes in the interactions between light-harvesting antennae and the photosynthetic reaction centers. Functional disconnection of antennae from PSII or PSI appears as a shift in the fluorescence peaks, because LHC antennae not connected to photosystems show a different fluorescence maximum than those connected (Wollman and Bennoun, 1982). An increase in the amplitude of the PSI peak illustrates a state 1-to-state 2 transition, which is the reversible transfer of a fraction of LHCII antenna from PSII to PSI (Wollman and Delepelaire, 1984).

Low-temperature fluorescence spectra have revealed that poor iron nutrition affects the light harvesting antennae associated with both photosystems (Moseley et al., 2002; Naumann et al., 2005). During iron-deficient photoheterotrophic growth, the amplitude of the LHCI/PSI peak increases and shifts toward 705 nm, which indicates a reduced energy transfer between the antennae and the reaction centers (higher amplitude) and a disconnection of LHCI antennae from PSI (shift). If the iron concentration in the media is further reduced (0.1 μM), the LHCII antennae are also disconnected from PSII as indicated by a shift of the 685 nm peak toward 680 nm (Moseley et al., 2002). In contrast, in the absence of acetate, the amplitude of the LCHI/PSI peak decreases, and there is only a small shift after extended iron-deplete growth (Busch et al., 2008).

Low temperature 77 K fluorescence has also been applied to characterize mutants involved in the iron deficiency response. In the *pgrl1-28* knock-down mutant for example, the antennae disconnection is more sensitive to iron-deficiency. An increase in amplitude and a small blue shift (from 713 to 709 nm) was observed for the mutant but not the wild-type at 3 μM Fe in the media (Petroutsos et al., 2009).

## Future direction

The role of iron in the metabolism of Chlamydomonas has been studied extensively; however, several questions remain to be investigated. Most iron nutrition studies have focused on growth in the presence of light and acetate. An outstanding question relates to the relationship between iron nutrition and carbon metabolism. Several studies have noted that the response of Chlamydomonas to iron status can vary drastically depending on whether acetate is present or not. Both the physiology as evaluated with growth kinetics, iron uptake and photosynthetic efficiency, and the transcriptome have revealed markedly different phenomena. How carbon source impacts the decision to maintain photosynthesis is not known. The relationship between Chlamydomonas iron metabolism and heterotrophic growth (in the dark with acetate) or anaerobiosis have been largely left uninvestigated.

How iron status is linked to changes in transcription is also unknown for Chlamydomonas. A number of transcription factors and regulatory proteins are known components of iron homeostasis in land plants (Vigani et al., 2013), but the extent to which there is overlap in the iron-regulatory networks between green algae and land plants is unknown. The recent iron-nutrition transcriptome of Chlamydomonas has provided some targets, which include eleven putative transcription regulators whose mRNA abundance is increased in iron-limited cells (Urzica et al., 2012). Of these putative regulators is a potential functional ortholog of the *A. thaliana* E3 ubiquitin ligase BTS and a bHLH transcription factor orthologous to bHLH115. However, as of yet, the functions of these putative regulators have not been confirmed in Chlamydomonas. Multiple types of iron-responsive elements (FeREs) have been uncovered (Deng and Eriksson, 2007; Fei et al., 2009, 2010), suggesting the involvement of more than one transcription factor. Also, the different responses to iron nutrition depending on carbon source suggest that the iron-regulatory network in Chlamydomonas may be complex.

### Conflict of interest statement

The authors declare that the research was conducted in the absence of any commercial or financial relationships that could be construed as a potential conflict of interest.

## References

[B1] Allen M. D. del Campo J. A. Kropat J. Merchant S. S. (2007a). *FEA1*, *FEA2*, and *FRE1*, encoding two homologous secreted proteins and a candidate ferrireductase, are expressed coordinately with *FOX1* and *FTR1* in iron-deficient *Chlamydomonas reinhardtii*. Eukaryot. Cell 6, 1841–1852 10.1128/EC.00205-07 17660359 PMC2043389

[B2] Allen M. D. Kropat J. Tottey S. del Campo J. A. Merchant S. S. (2007b). Manganese deficiency in Chlamydomonas results in loss of photosystem II and MnSOD function, sensitivity to peroxides, and secondary phosphorus and iron deficiency. Plant Physiol. 143, 263–277 10.1104/pp.106.088609 17085511 PMC1761973

[B3] Baker N. R. (2008). Chlorophyll fluorescence: a probe of photosynthesis *in vivo*. Annu. Rev. Plant Biol. 59, 89–113 10.1146/annurev.arplant.59.032607.092759 18444897

[B4] Becker B. (2013). Snow ball earth and the split of Streptophyta and Chlorophyta. Trends Plant Sci. 18, 180–183 23102566 10.1016/j.tplants.2012.09.010

[B5] Ben-Shem A. Frolow F. Nelson N. (2003). Crystal structure of plant photosystem I. Nature 426, 630–635 10.1038/nature02200 14668855

[B6] Blaby-Haas C. E. Merchant S. S. (2013). Sparing and salvaging metals in chloroplasts, in Encyclopedia of Inorganic and Bioinorganic Chemistry. Metals in Cells, eds Culotta V. Scott R. A. (Chichester: John Wiley and Sons Ltd) (in press).

[B7] Brown J. (1956). Iron chlorosis. Ann. Rev. Plant Physiol. 7, 171–190

[B8] Buckhout T. J. Yang T. J. W. Schmidt W. (2009). Early iron-deficiency-induced transcriptional changes in *Arabidopsis* roots as revealed by microarray analyses. BMC Genomics 10:147 10.1186/1471-2164-10-147 19348669 PMC2676303

[B9] Busch A. Rimbauld B. Naumann B. Rensch S. Hippler M. (2008). Ferritin is required for rapid remodeling of the photosynthetic apparatus and minimizes photo-oxidative stress in response to iron availability in *Chlamydomonas reinhardtii*. Plant J. 55, 201–211 10.1111/j.1365-313X.2008.03490.x 18363784

[B10] Chen Y. Barak P. (1982). Iron nutrition of plants in calcareous soils. Adv. Agron. 35, 217–240

[B11] Cox C. D. (1994). Deferration of laboratory media and assays for ferric and ferrous ions. Methods Enzymol. 235, 315–329 10.1016/0076-687935150-3 8057904

[B12] Cvetkovic A. Menon A. L. Thorgersen M. P. Scott J. W. Poole F. L. 2nd. Jenney F. E. Jr. (2010). Microbial metalloproteomes are largely uncharacterized. Nature 466, 779–782 10.1038/nature09265 20639861

[B13] Deng X. Eriksson M. (2007). Two iron-responsive promoter elements control expression of FOX1 in *Chlamydomonas reinhardtii*. Eukaryot. Cell 6, 2163–2167 10.1128/EC.00324-07 17905921 PMC2168406

[B14] Dix D. R. Bridgham J. T. Broderius M. A. Byersdorfer C. A. Eide D. J. (1994). The *FET4* gene encodes the low affinity Fe(II) transport protein of *Saccharomyces cerevisiae*. J. Biol. Chem. 269, 26092–26099 7929320

[B15] Docampo R. de Souza W. Miranda K. Rohloff P. Moreno S. N. (2005). Acidocalcisomes - conserved from bacteria to man. Nat. Rev. Microbiol. 3, 251–261 10.1038/nrmicro1097 15738951

[B16] Eide D. Broderius M. Fett J. Guerinot M. L. (1996). A novel iron-regulated metal transporter from plants identified by functional expression in yeast. Proc. Natl. Acad. Sci. U.S.A. 93, 5624–5628 8643627 10.1073/pnas.93.11.5624 PMC39298

[B17] Emerson L. R. Nau M. E. Martin R. K. Kyle D. E. Vahey M. Wirth D. F. (2002). Relationship between chloroquine toxicity and iron acquisition in *Saccharomyces cerevisiae*. Antimicrob. Agents Chemother. 46, 787–796 10.1128/AAC.46.3.787-796.2002 11850263 PMC127479

[B18] Fei X. Eriksson M. Li Y. Deng X. (2010). A novel negative fe-deficiency-responsive element and a TGGCA-type-like FeRE control the expression of FTR1 in *Chlamydomonas reinhardtii*. J. Biomed. Biotechnol. 2010:790247 10.1155/2010/790247 20182641 PMC2826095

[B19] Fei X. Eriksson M. Yang J. Deng X. (2009). An Fe deficiency responsive element with a core sequence of TGGCA regulates the expression of FEA1 in *Chlamydomonas reinharditii*. J. Biochem. 146, 157–166 10.1093/jb/mvp056 19351705

[B20] Fischer B. B. Wiesendanger M. Eggen R. I. L. (2006). Growth condition-dependent sensitivity, photodamage and stress response of *Chlamydomonas reinhardtii* exposed to high light conditions. Plant Cell Physiol. 47, 1135–1145 10.1093/pcp/pcj085 16857695

[B21] Giehl R. F. H. Lima J. E. von Wirén N. (2012). Localized iron supply triggers lateral root elongation in *Arabidopsis* by altering the AUX1-mediated Auxin distribution. Plant cell 24, 33–49 10.1105/tpc.111.092973 22234997 PMC3289578

[B22] Grossman A. R. Croft M. Gladyshev V. N. Merchant S. S. Posewitz M. C. Prochnik S. (2007). Novel metabolism in Chlamydomonas through the lens of genomics. Curr. Opin. Plant Biol. 10, 190–198 10.1016/j.pbi.2007.01.012 17291820

[B23] Herbik A. Bölling C. Buckhout T. J. (2002). The involvement of a multicopper oxidase in iron uptake by the green algae *Chlamydomonas reinhardtii*. Plant Physiol. 130, 2039–2048 10.1104/pp.013060 12481087 PMC166715

[B24] Hong S. Kim S. A. Guerinot M. L. McClung C. R. (2013). Reciprocal interaction of the circadian clock with the iron homeostasis network in Arabidopsis. Plant Physiol. 161, 893–903 10.1104/pp.112.208603 23250624 PMC3561027

[B25] Hou X. Jones B. T. (2000). Inductively coupled plasma-optical emission spectrometry, in Encyclopedia of Analytical Chemistry, ed Meyers R. A. (Chichester: John Wiley and Sons Ltd.), 9468–9485

[B26] Husted S. Persson D. P. Laursen K. H. Hansen T. H. Pedas P. Schiller M. (2011). The role of atomic spectrometry in plant science. J. Anal. At. Spectrom. 26, 52–79 10.1039/c0ja00058b

[B27] Hutner S. H. Provasoli L. Schatz A. Haskins C. P. (1950). Some approaches to the study of the role of metals in the metabolism of microorganisms. Proc. Am. Phil. Soc. 94, 152–170 919901

[B28] Jacobs J. Pudollek S. Hemschemeier A. Happe T. (2009). A novel, anaerobically induced ferredoxin in *Chlamydomonas reinhardtii*. FEBS Lett. 583, 325–329 10.1016/j.febslet.2008.12.018 19101555

[B29] Jo W. J. Kim J. H. Oh E. Jaramillo D. Holman P. Loguinov A. V. (2009). Novel insights into iron metabolism by integrating deletome and transcriptome analysis in an iron deficiency model of the yeast *Saccharomyces cerevisiae*. BMC Genomics 10:130 10.1186/1471-2164-10-130 19321002 PMC2669097

[B30] Kroll H. Knell M. Powers J. Simonian J. (1957). A phenolic analog of ethylenediamine-tetraacetic acid. J. Am. Chem. Soc. 79, 2024–2025 10.1021/ja01565a075

[B31] Kropat J. Hong-Hermesdorf A. Casero D. Ent P. Castruita M. Pellegrini M. (2011). A revised mineral nutrient supplement increases biomass and growth rate in *Chlamydomonas reinhardtii*. Plant J. 66, 770–780 10.1111/j.1365-313X.2011.04537.x 21309872 PMC3101321

[B32] La Fontaine S. Quinn J. M. Nakamoto S. S. Page M. D. Göhre V. Moseley J. L. (2002). Copper-dependent iron assimilation pathway in the model photosynthetic eukaryote *Chlamydomonas reinhardtii*. Eukaryot. Cell 1, 736–757 10.1128/EC.1.5.736-757.2002 12455693 PMC126744

[B33] Lanquar V. Lelièvre F. Bolte S. Hamès C. Alcon C. Neumann D. (2005). Mobilization of vacuolar iron by AtNRAMP3 and AtNRAMP4 is essential for seed germination on low iron. EMBO J. 24, 4041–4051 10.1038/sj.emboj.7600864 16270029 PMC1356305

[B34] Lesuisse E. Simon-Casteras M. Labbe P. (1998). Siderophore-mediated iron uptake in *Saccharomyces cerevisiae*: the *SIT1* gene encodes a ferrioxamine B permease that belongs to the major facilitator superfamily. Microbiology 144, 3455–3462 10.1099/00221287-144-12-3455 9884238

[B35] Long J. C. Merchant S. S. (2008). Photo-oxidative stress impacts the expression of genes encoding iron metabolism components in Chlamydomonas. Photochem. Photobiol. 84, 1395–1403 10.1111/j.1751-1097.2008.00451.x 19067961

[B36] Long J. C. Sommer F. Allen M. D. Lu S. F. Merchant S. S. (2008). *FER1* and *FER2* encoding two ferritin complexes in *Chlamydomonas reinhardtii* chloroplasts are regulated by iron. Genetics 179, 137–147 10.1534/genetics.107.083824 18493046 PMC2390593

[B37] Lynnes J. A. Derzaph T. L. M. Weger H. G. (1998). Iron limitation results in induction of ferricyanide reductase and ferric chelate reductase activities in *Chlamydomonas reinhardtii*. Planta 204, 360–365 10.1007/s004250050267

[B38] Malasarn D. Kropat J. Hsieh S. I. Finazzi G. Casero D. Loo J. A. (2013). Zinc deficiency impacts CO_2_ assimilation and disrupts copper homeostasis in *Chlamydomonas reinhardtii*. J. Biol. Chem. 288, 10672–10683 10.1074/jbc.M113.455105 23439652 PMC3624447

[B39] Matsumura K. Yagi T. Yasuda K. (2003). Role of timer and sizer in regulation of *Chlamydomonas* cell cycle. Biochem. Biophys. Res. Commun. 306, 1042–1049 10.1016/S0006-291X(03)01089-1 12821148

[B40] Maxwell K. Johnson G. N. (2000). Chlorophyll fluorescence - a practical guide. J. Exp. Bot. 51, 659–668 10.1093/jexbot/51.345.659 10938857

[B41] Mengel K. (1994). Iron availability in plant tissues - iron chlorosis on calcareous soils. Plant Soil 165, 275–283 10.1007/BF00008070

[B42] Merchant S. S. Allen M. D. Kropat J. Moseley J. L. Long J. C. Tottey S. (2006). Between a rock and a hard place: trace element nutrition in Chlamydomonas. Biochim. Biophys. Acta 1763, 578–594 10.1016/j.bbamcr.2006.04.007 16766055

[B43] Mercier A. Pelletier B. Labbé S. (2006). A Transcription factor cascade involving Fep1 and the CCAAT-binding factor Php4 regulates gene expression in response to iron deficiency in the fission yeast *Schizosaccharomyces pombe*. Eukaryot. Cell 5, 1866–1881 10.1128/EC.00199-06 16963626 PMC1694796

[B44] Moore J. K. Doney S. C. Glover D. M. Fung I. Y. (2002). Iron cycling and nutrient-limitation patterns in surface waters of the World Ocean. Deep Sea Res. II 49, 463–507 10.1016/S0967-064500109-6

[B45] Moseley J. L. Allinger T. Herzog S. Hoerth P. Wehinger E. Merchant S. (2002). Adaptation to Fe-deficiency requires remodeling of the photosynthetic apparatus. EMBO J. 21, 6709–6720 10.1093/emboj/cdf666 12485992 PMC139087

[B46] Moseley J. L. Quinn J. Eriksson M. Merchant S. (2000). The *Crd1* gene encodes a putative di-iron enzyme required for photosystem I accumulation in copper deficiency and hypoxia in *Chlamydomonas reinhardtii*. EMBO J. 19, 2139–2151 10.1093/emboj/19.10.2139 10811605 PMC384357

[B47] Murakami A. Fujita Y. Nemson J. A. Melis A. (1997). Chromatic regulation in *Chlamydomonas reinhardtii*: time course of photosystem stoichiometry adjustment following a shift in growth light quality. Plant Cell Physiol. 38, 188–193

[B48] Murata N. (1968). Fluorescence of chlorophyll in photosynthetic systems. IV. Induction of various emissions at low temperatures. Bioenergetics 162, 106–121 10.1016/0005-272890219-3 5665255

[B49] Nagasaka S. Yoshimura E. (2008). External iron regulates polyphosphate content in the acidophilic, thermophilic alga *Cyanidium caldarium*. Biol. Trace Elem. Res. 125, 286–289 10.1007/s12011-008-8177-9 18575816

[B50] Naumann B. Busch A. Allmer J. Ostendorf E. Zeller M. Kirchhoff H. (2007). Comparative quantitative proteomics to investigate the remodeling of bioenergetic pathways under iron deficiency in *Chlamydomonas reinhardtii.* Proteomics 7, 3964–3979 10.1002/pmic.200700407 17922516

[B51] Naumann B. Stauber E. J. Busch A. Sommer F. Hippler M. (2005). N-terminal processing of Lhca3 is a key step in remodeling of the photosystem I-light-harvesting complex under iron deficiency in *Chlamydomonas reinhardtii*. J. Biol. Chem. 280, 20431–20441 10.1074/jbc.M414486200 15774469

[B52] Niemelä M. Perämäki P. Kola H. Piispanen J. (2003). Determination of arsenic, iron and selenium in moss samples using hexapole collision cell, inductively coupled plasma-mass spectrometry. Anal. Chim. Acta 493, 3–12 10.1016/S0003-267000819-5

[B53] Page M. D. Allen M. D. Kropat J. Urzica E. I. Karpowicz S. J. Hsieh S. I. (2012). Fe sparing and Fe recycling contribute to increased superoxide dismutase capacity in iron-starved *Chlamydomonas reinhardtii*. Plant Cell 24, 2649–2665 10.1105/tpc.112.098962 22685165 PMC3406916

[B54] Page M. D. Kropat J. Hamel P. P. Merchant S. S. (2009). Two *Chlamydomonas* CTR copper transporters with a novel cys-met motif are localized to the plasma membrane and function in copper assimilation. Plant Cell 21, 928–943 10.1105/tpc.108.064907 19318609 PMC2671701

[B55] Paz Y. Shimoni E. Weiss M. Pick U. (2007). Effects of iron deficiency on iron binding and internalization into acidic vacuoles in *Dunaliella salina*. Plant Physiol. 144, 1407–1415 10.1104/pp.107.100644 17513481 PMC1914149

[B56] Pelletier B. Trott A. Morano K. A. Labbé S. (2005). Functional characterization of the iron-regulatory transcription factor Fep1 from *Schizosaccharomyces pombe*. J. Biol. Chem. 280, 25146–25161 10.1074/jbc.M502947200 15866870

[B57] Petroutsos D. Terauchi A. M. Busch A. Hirschmann I. Merchant S. S. Finazzi G. (2009). PGRL1 participates in iron-induced remodeling of the photosynthetic apparatus and in energy metabolism in *Chlamydomonas reinhardtii*. J. Biol. Chem. 284, 32770–32781 10.1074/jbc.M109.050468 19783661 PMC2781694

[B58] Porra R. J. Thompson W. A. Kriedemann P. E. (1989). Determination of accurate extinction coefficients and simultaneous equations for assaying chlorophylls *a* and *b* extracted with four different solvents: verification of the concentration of chlorophyll standards by atomic absorption spectroscopy. Bioenergetics 975, 384–394 10.1016/S0005-272880347-0

[B59] Pröfrock D. Prange A. (2012). Inductively Coupled Plasma–Mass Spectrometry (ICP-MS) for quantitative analysis in environmental and life sciences: a review of challenges, solutions, and trends. Appl. Spectrosc. 66, 843–868 10.1366/12-06681 22800465

[B60] Quinn J. M. Merchant S. S. (1998). Copper-responsive gene expression during adaptation to copper deficiency. Methods Enzymol. 297, 263–279 10.1016/S0076-687997020-687997023 9750208

[B61] Rochaix J.-D. (2002). *Chlamydomonas*, a model system for studying the assembly and dynamics of photosynthetic complexes. FEBS Lett. 529, 34–38 10.1016/S0014-579303181-2 12354609

[B62] Rubinelli P. Siripornadulsil S. Gao-Rubinelli F. Sayre R. T. (2002). Cadmium- and iron-stress-inducible gene expression in the green alga *Chlamydomonas reinhardtii*: evidence for H43 protein function in iron assimilation. Planta 215, 1–13 10.1007/s00425-001-0711-3 12012236

[B63] Ruiz F. A. Marchesini N. Seufferheld M. Govindjee, Docampo R. (2001). The polyphosphate bodies of *Chlamydomonas reinhardtii* possess a proton-pumping pyrophosphatase and are similar to acidocalcisomes. J. Biol. Chem. 276, 46196–46203 10.1074/jbc.M105268200 11579086

[B64] Salomé P. A. Oliva M. Weigel D. Krämer U. (2013). Circadian clock adjustment to plant iron status depends on chloroplast and phytochrome function. EMBO J. 32, 511–523 10.1038/emboj.2012.330 23241948 PMC3579136

[B65] Semin B. K. Davletshina L. N. Novakova A. A. Kiseleva T. Y. Lanchinskaya V. Y. Aleksandrov A. Y. (2003). Accumulation of ferrous iron in *Chlamydomonas reinhardtii*. Influence of CO_2_ and anaerobic induction of the reversible hydrogenase. Plant Physiol. 131, 1756–1764 10.1104/pp.102.018200 12692334 PMC166931

[B66] Spiller S. C. Castelfranco A. M. Castelfranco P. A. (1982). Effects of iron and oxygen on chlorophyll biosynthesis I. *In vivo* observations on iron and oxygen-deficient plants. Plant Physiol. 69, 107–111 10.1104/pp.69.1.107 16662138 PMC426155

[B67] Stookey L. L. (1970). Ferrozine - a new spectrophotometric reagent for iron. Anal. Chem. 42, 779–781 10.1021/ac60289a016

[B68] Terauchi A. M. Peers G. Kobayashi M. C. Niyogi K. K. Merchant S. S. (2010). Trophic status of *Chlamydomonas reinhardtii* influences the impact of iron deficiency on photosynthesis. Photosynth. Res. 105, 39–49 10.1007/s11120-010-9562-8 20535560 PMC2885298

[B69] Terzulli A. Kosman D. J. (2010). Analysis of the high-affinity iron uptake system at the *Chlamydomonas reinhardtii* plasma membrane. Eukaryot. Cell 9, 815–826 10.1128/EC.00310-09 20348389 PMC2863958

[B70] Thimm O. Essigmann B. Kloska S. Altmann T. Buckhout T. J. (2001). Response of Arabidopsis to iron deficiency stress as revealed by microarray analysis. Plant Physiol. 127, 1030–1043 10.1104/pp.010191 11706184 PMC129273

[B71] Tottey S. Block M. A. Allen M. Westergren T. Albrieux C. Scheller H. V. (2003). *Arabidopsis* CHL27, located in both envelope and thylakoid membranes, is required for the synthesis of protochlorophyllide. Proc. Natl. Acad. Sci. U.S.A. 100, 16119–16124 10.1073/pnas.2136793100 14673103 PMC307702

[B72] Umen J. D. (2005). The elusive sizer. Curr. Opin. Cell Biol. 17, 435–441 10.1016/j.ceb.2005.06.001 15978795

[B73] Urzica E. I. Casero D. Yamasaki H. Hsieh S. I. Adler L. N. Karpowicz S. J. (2012). Systems and *trans*-system level analysis identifies conserved iron deficiency responses in the plant lineage. Plant Cell 24, 3921–3948 10.1105/tpc.112.102491 23043051 PMC3517228

[B74] Vance P. Spalding M. H. (2005). Growth, photosynthesis, and gene expression in *Chlamydomonas* over a range of CO_2_ concentrations and CO_2_/O_2_ ratios: CO_2_ regulates multiple acclimation states. Can. J. Bot. 83, 796–809 10.1139/b05-064

[B75] Vert G. Grotz N. Dédaldéchamp F. Gaymard F. Guerinot M. L. Briat J.-F. (2002). IRT1, an arabidopsis transporter essential for iron uptake from the soil and for plant growth. Plant Cell 14, 1223–1233 10.1105/tpc.001388 12084823 PMC150776

[B76] Vigani G. Zocchi G. Bashir K. Philippar K. Briat J.-F. (2013). Signals from chloroplasts and mitochondria for iron homeostasis regulation. Trends Plant Sci. 8, 305–311 10.1016/j.tplants.2013.01.006 23462548

[B77] Vogl J. Klingbeil P. Pritzkow W. Riebe G. (2003). High accuracy measurements of Fe isotopes using hexapole collision cell MC-ICP-MS and isotope dilution for certification of reference materials. J. Anal. Atom. Spectrom. 18, 1125–1132 10.1039/B301812A

[B78] Wang H.-Y. Klatte M. Jakoby M. Bäumlein H. Weisshaar B. Bauer P. (2007). Iron deficiency-mediated stress regulation of four subgroup Ib *BHLH* genes in *Arabidopsis thaliana*. Planta 226, 897–908 10.1007/s00425-007-0535-x 17516080

[B79] Weger H. G. (1999). Ferric and cupric reductase activities in the green alga *Chlamydomonas reinhardtii*: Experiments using iron-limited chemostats. Planta 207, 377–384 10.1007/s004250050495

[B80] Weger H. G. Espie G. S. (2000). Ferric reduction by iron-limited *Chlamydomonas* cells interacts with both photosynthesis and respiration. Planta 210, 775–781 10.1007/s004250050679 10805449

[B81] Wollman F.-A. Bennoun P. (1982). A new chlorophyll-protein complex related to photosystem I in *Chlamydomonas reinhardii*. Bioenergetics 680, 352–360 10.1016/0005-272890149-92834098

[B82] Wollman F.-A. Delepelaire P. (1984). Correlation between changes in light energy distribution and changes in thylakoid membrane polypeptide phosphorylation in *Chlamydomonas reinhardtii*. J. Cell Biol. 98, 1–7 10.1083/jcb.98.1.1 6707079 PMC2113001

[B83] Xu W. Barrientos T. Andrews N. C. (2013). Iron and copper in mitochondrial diseases. Cell Metab. 17, 319–328 10.1016/j.cmet.2013.02.004 23473029 PMC3594794

[B84] Xue X. Collins C. M. Weger H. G. (1998). The energetics of extracellular Fe(III) reduction by iron-limited *Chlamydomonas reinhardtii* (Chlorophyta). J. Phycol. 34, 939–944 10.1046/j.1529-8817.1998.340939.x10805449

[B85] Yang T. J. W. Lin W.-D. Schmidt W. (2010). Transcriptional profiling of the Arabidopsis iron deficiency response reveals conserved transition metal homeostasis networks. Plant Physiol. 152, 2130–2141 10.1104/pp.109.152728 20181752 PMC2850031

[B86] Yip Y.-c. Sham W.-c. (2007). Application of collision/reaction-cell technology in isotope dilution mass spectrometry. Trends Anal. Chem. 26, 727–743 10.1016/j.trac.2007.03.007

[B87] Zhang L. Happe T. Melis A. (2002). Biochemical and morphological characterization of sulfur-deprived and H2-producing *Chlamydomonas reinhardtii* (green alga). Planta 214, 552–561 10.1007/s004250100660 11925039

[B88] Zheng L. Huang F. Narsai R. Wu J. Giraud E. He F. (2009). Physiological and transcriptome analysis of iron and phosphorus interaction in rice seedlings. Plant Physiol. 151, 262–274 10.1104/pp.109.141051 19605549 PMC2735995

